# Altered Gravity Simulated by Parabolic Flight and Water Immersion Leads to Decreased Trunk Motion

**DOI:** 10.1371/journal.pone.0133398

**Published:** 2015-07-24

**Authors:** Peiliang Wang, Zheng Wang, Dongni Wang, Yu Tian, Fan Li, Shaoyao Zhang, Lin Zhang, Yaoyu Guo, Weibo Liu, Chunhui Wang, Shanguang Chen, Jinhu Guo

**Affiliations:** 1 Key Laboratory of Gene Engineering of the Ministry of Education, State Key Laboratory of Biocontrol, School of Life Sciences, Sun Yat-sen University, Guangzhou, China; 2 National Key Laboratory of Human Factors Engineering, China Astronaut Research and Training Center, Beijing, China; Charles P. Darby Children's Research Institute, UNITED STATES

## Abstract

Gravity is one of the important environmental factors that influence the physiologies and behaviors of animals and humans, and changes in gravity elicit a variety of physiological and behavioral alterations that include impaired movement coordination, vertigo, spatial disorientation, and perceptual illusions. To elucidate the effects of gravity on human physiology and behavior, we examined changes in wrist and trunk activities and heart rate during parabolic flight and the activity of wrist and trunk in water immersion experiments. Data from 195 person-time parabolas performed by eight subjects revealed that the trunk motion counts decreased by approximately half during ascending legs (hypergravity), relative to the data acquired before the parabolic flights. In contrast, the wrist activity remained unchanged. The results from the water immersion experiments demonstrated that in the underwater condition, both the wrist and trunk activities were significantly decreased but the latter decreased to a much lower level. Together, these data suggest that gravitational alterations can result in differential influences on the motions of the wrist and the trunk. These findings might be important for understanding the degeneration of skeleton and muscular system and performance of astronauts in microgravity.

## Introduction

All lives on the Earth have evolved and developed in the constant presence of 1.0*g* gravity [1]. To study the changes in physiology and behavior that occur in microgravity, a number of approaches that are applied on the ground have been used to create simulated weightlessness, including head-down bed rest (HDBR), parabolic flight and water immersion [2–4]. Microgravity can be simulated by free-fall during parabolic flights, which follow the ballistic trajectory of a parabola. Parabolic flights produce short successive periods of altered gravity within the range 0 and 1.8*g* [5]. Water immersion is another approach creating conditions of weight compensation and allows for the study of performance in hypogravity conditions [6, 7].

Changes in gravity have deleterious effects on physiology and behavior that lead to disorientation, muscular atrophy and bone mass loss and deregulation of circadian rhythms and sleep [8–13]. Additionally alterations in gravity influences cognitive performance [1,14]. When exposed to unusual gravity environments, subjects produce exaggerated isometric forces [3,15]. A comparison of muscle activities during walking underwater and on land at a slow speed revealed that activities of the rectus abdominis and paraspinal muscles remained unchanged, but the activities of all of the other tested muscles, including gluteus medius, rectus femoris, vastusmedialis, biceps femoris andtibialis anterior gastrocnemius, decreased underwater [16]. These facts suggest that alterations in gravity affect motion and performance.

In a previous study, we found that trunk motions of two orbital astronauts decreased dramatically during a space mission [17], suggesting that microgravity might impose specific effects on trunk motion. Access to space experiments is very expensive and limited, and the number of subjects is typically low. As such, some of the observations obtained from space study require validation in ground-based simulations. In the present work, we analyzed data from parabolic flights and water immersion experiments to further address the influence of simulated gravitational alterations on trunk and wrist motions. The findings of this work may help to facilitate the development of more effective countermeasures for astronauts.

## Materials and Methods

### Parabolic flights

**Fig 1:**
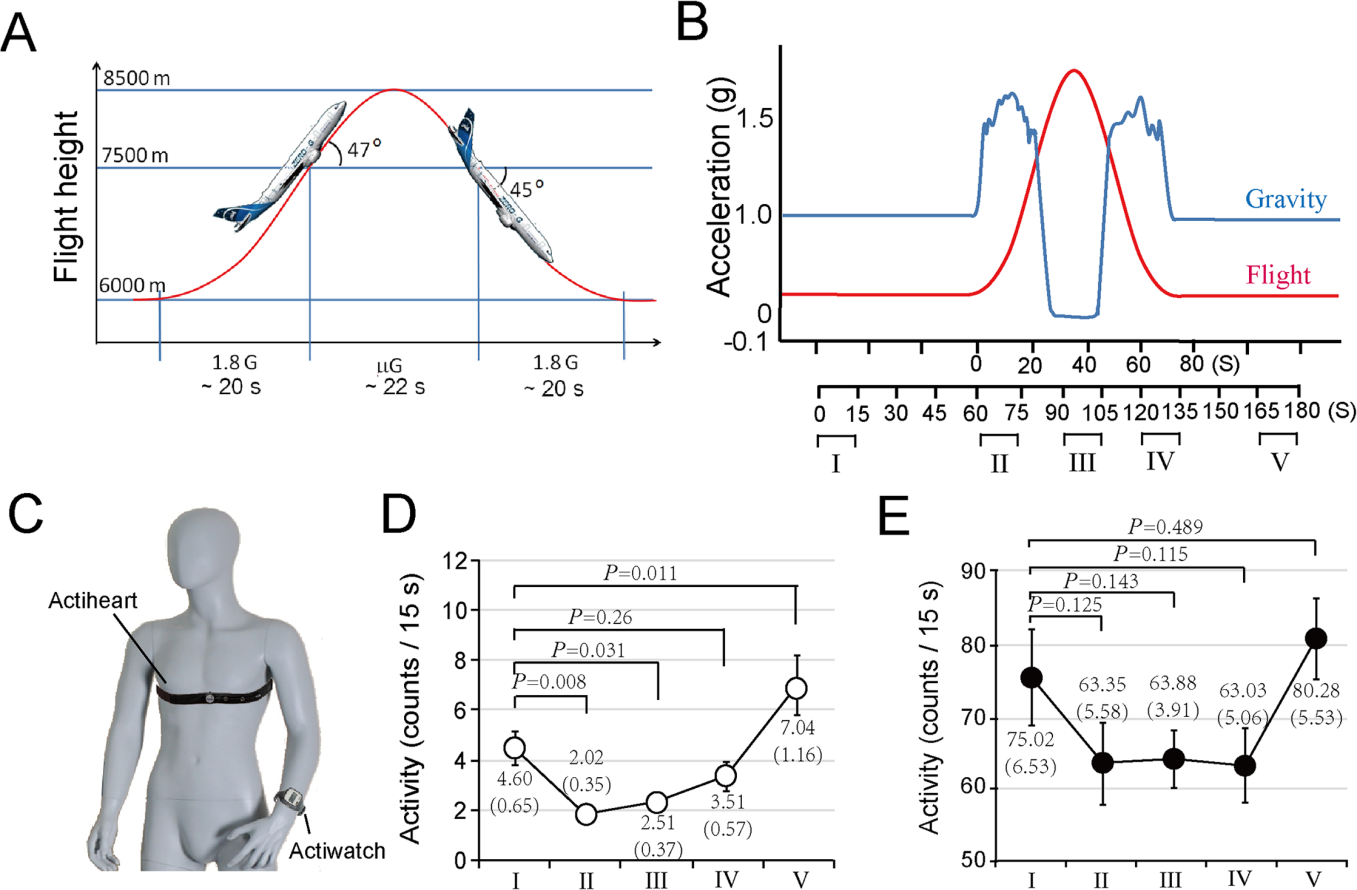
Influences of parabolic flight on activity of wrist and trunk activities. (A) Schematic representation of the parabolic flight profile. (B) The gravity levels that occurred during the parabolic flights and the blocks used for data collection (I-V). Block I: prior to parabola; block II: during hypergravity; block III: during hypogravity; block IV: during hypergravity; block V: prior after parabola. The duration of each block was 15 s. (C) The placements of Actiheart and Actiwatch on a human subject. (D) Trunk activity recorded by the Actiheart. The data are presented as the averages of 195 parabolas completed by seven subjects. (E) Wrist activity recorded by Actiwatch. Data are averages from the mean values of eight subjects. Statistical significance was determined with one-way repeated-measures ANOVA with Fisher’s least significance difference (LSD) post hoc test. The data are means ± SEs. The *P* values are shown to indicate significant differences.

The 112^th^ parabolic flight campaign was organized by ESA and NOVESPACE at the Société Girondine ďEquipments, de Réparation, et de Maintenance Aéronautique (SOGERMA) center in Bordeaux, France. This study was conducted from Oct. 7 –Oct. 9 in 2014. The AIRBUS A300 ZERO-G aircraft was employed to perform the parabolic flights. Each flight consisted of 31 parabolas. Data from the following five phases (blocks) of each parabola (termed I–V) were sampled and analyzed. I, 1 *g*, approximately 45 s before the parabolic flights; II, ~1.8 *g*, during the ascending leg (hypergravity); III, μ*g* at the apex (microgravity); IV, ~1.8 *g* during the descending leg (hypergravity) and V, 1 *g*, approximately 30 s before parabolic flights (Fig 1A and 1B). The subjects were trained to assume supine or squatting positions in hypergravity and were free to move during other periods.

An Actiwatch was used to record the wrist activity, and an Actiheart to record the heart rate (HR) variables and trunk activity. The Actiheart was worn around the chest with a belt according to the manufacturer’s instruction (Fig 1C). To prevent the Actiheart worn with a chest belt from getting loose owing to movement, it was tightened with a bandage around the chest. These devices were worn before take-off and removed for analysis after landing.

### Water immersion experiments

The water immersion experiments were performed in the China Astronaut Research and Training Center in Beijing, China, from 2015 Jan 9 to Feb 4. The room temperature was 26 ± 2°C, and the water temperature was 30 ± 2°C. The volunteers were completing regular trainings that include diving, moving objects vertically and horizontally and videotaping. The data that were recorded for approximately 30 min on the land and equivalent underwater time were subject to analysis.

Actiwatchs sealed in double-layered plastic bags were worn on both the wrist and the chest to simultaneously record activity data. A bandage was wrapped around the chest to fix the Actiwatch.

### Subjects

Each flight consists of approximately 30 parabolic cycles. Some of the subjects participated in more than one flight. Invalid data from some of the parabolas were discarded. There were eight male volunteers who were between the ages of 26 and 44 years (mean 32.8yrs). In total the volunteers participated in 248 person-time parabolas. All of the volunteers passed the physical tests for the Aeronautical Medical Certification carried out by authorized hospitals in China or France. All of the subjects underwent extensive physical examinations and were free of all cardiovascular, metabolic and neural pathology diseases. All but one of the subjects took prophylactic medication (i.e., injected of scopolamine) before boarding the plane, and none of the subjects showed symptoms of motion sickness during the flights. Eleven male volunteers between the ages of 23 and 40 years (mean 29.5 yrs), participated in 22 person-time water immersion experiments.

All experimental protocols were approved by the Ethics Committee of the China Astronaut Research and Training Center. The subjects were informed of the potential physiological and mental consequences of undergoing prolonged bed rest, and consent forms were signed. All of the subjects were required to provide written informed consent prior to their participation in the study.

### Equipment

Ambulatory 'Actiwatch AW2' monitors (Camntech Ltd., UK) was used to monitor the activity parameters. The Actiwatch records and assesses body movement by integrating the degree and speed of the movement that occurs within a given epoch. In the present work, the epoch of Actiwatch was set to 15 s. All of the Actiwatchs were calibrated to record with an accuracy of exceeding 95% by the manufacturer. The Actiwatch data were analyzed with Actiware software (V5.70).

Actiheart monitors (CamntechLtd., UK) were used to collect data of heart rate and trunk data during the parabolic flights. The Actiheart is a device that combines a HR monitor and a movement sensor into a single unit. A number of studies have demonstrated the accuracy and reliability of the Actiheart [18–20]. According to the manual, the Actihearts were calibrated thoroughly on a specially designed rig by the manufacturer. The Actiheart incorporates a calibration factor to produce the same output for the same amount of motion (in terms of g).In this study, the Actiheart was set up to simultaneously record heart beat, activity and the interbeat interval (IBI), including Minimum and maximum values of IBI in the epoch. The recording epoch was 15s. The data acquired with the Actiheart were analyzed with the Actiheart software (V4.0.100). All heart rate and activity were recorded simultaneously, and data that included aberrant heart rate values (e.g., 0) were considered non-useable and eliminated prior to the analysis. For the parabolic study, the times of Actihearts and Actiwatchs were synchronized to the coordinated universal time (UTC) time displayed on the plane. The time used in the water immersion experiments was synchronized to the local time.

### Statistics

The results are presented as means± SEs. Statistical significance was determined with one-way analyses of variance (ANOVA) with Fisher’s least significance difference (LSD) post hoc tests for repeated measurements or Student-*t* tests as indicated. The *P* values are shown in the figures or tables.

## Results

### The influence of parabolic flight on activity

Actihearts were worn to record the heart rate and trunk activity data, and Actiwatchs were used to record wrist activity data. Of all the 248 person-time parabolas, the data that contained unreasonable heart rate values (e.g., 0) due to the loosening of the belts holding the Actihearts were regarded as invalid and precluded for analysis. In total, the data from 195 validated person-time parabolas were valid. We analyzed the data from all of the phases (I-V), and the results revealed an U-shaped profile of both the wrist and trunk activity changes (Fig 1D and 1E, Table 1). Compared with phase I, the trunk activity was significantly decreased in phases II and III, unchanged in phase IV, and significantly increased at phase V (Fig 1D). In contrast, the wrist activity revealed no significant change across the phases, although the changes displayed an U-shaped tendency (Fig 1E).

**Table 1:** Changes in wrist and trunk the activities during the parabolic flights.

Subjects	Trunk motion					Wrist motion				
	Parabolic phases					Parabolic phases				
	I	II	III	IV	V	I	II	III	IV	V
1	6.67	8.67	3.90	9.43	14.10	75.80	113.93	74.43	80.63	102.97
	(2.03)	(1.62)	(1.46)	(2.25)	(3.26)	(13.76)	(22.40)	(11.45)	(14.96)	(12.93)
2	2.20	0.47	5.23	0.63	3.33	39.87	55.47	81.83	60.43	57.77
	(0.41)	(0.09)	(0.97)	(0.12)	(0.62)	(7.40)	(10.30)	(15.20)	(11.22)	(10.73)
3	3.93	0.33	1.77	2.47	4.37	49.20	15.20	51.10	35.20	44.40
	(1.17)	(0.16)	(1.08)	(1.30)	(1.86)	(13.32)	(4.15)	(9.72)	(9.30)	(11.87)
4	10.17	1.07	0.27	5.30	17.33	146.57	111.37	58.93	94.80	140.87
	(2.53)	(0.40)	(0.74)	(1.82)	(5.18)	(25.31)	(19.08)	(8.59)	(14.705)	(15.32)
5	2.57	1.48	2.67	2.00	3.52	61.00	55.24	95.52	68.48	76.14
	(1.43)	(0.56)	(0.72)	(0.88)	(1.91)	(12.53)	(9.13)	(13.86)	(13.13)	(15.04)
6	4.83	1.39	0.35	4.35	4.26	105.39	60.26	58.48	62.17	102.13
	(1.82)	(0.57)	(0.15)	(1.52)	(2.20)	(22.39)	(5.84)	(11.50)	(11.90)	(18.20)
7	1.56	0.56	3.30	0.26	1.00	54.59	30.41	37.63	44.82	42.19
	(0.73)	(0.25)	(0.52)	(0.19)	(0.78)	(11.28)	(2.23)	(5.04)	(5.23)	(9.04)
8	0.00	0.00	0.25	0.00	0.00	26.50	26.75	54.59	4.358	47.00
	(0.00)	(0.00)	(0.25)	(0.00)	(0.00)	(26.50)	(9.69)	(17.45)	(1.52)	(28.47)

The data are represented as the average of trunk and wrist activity counts during all the parabolas of each subject. Data are means (SEs).

**Fig 2:**
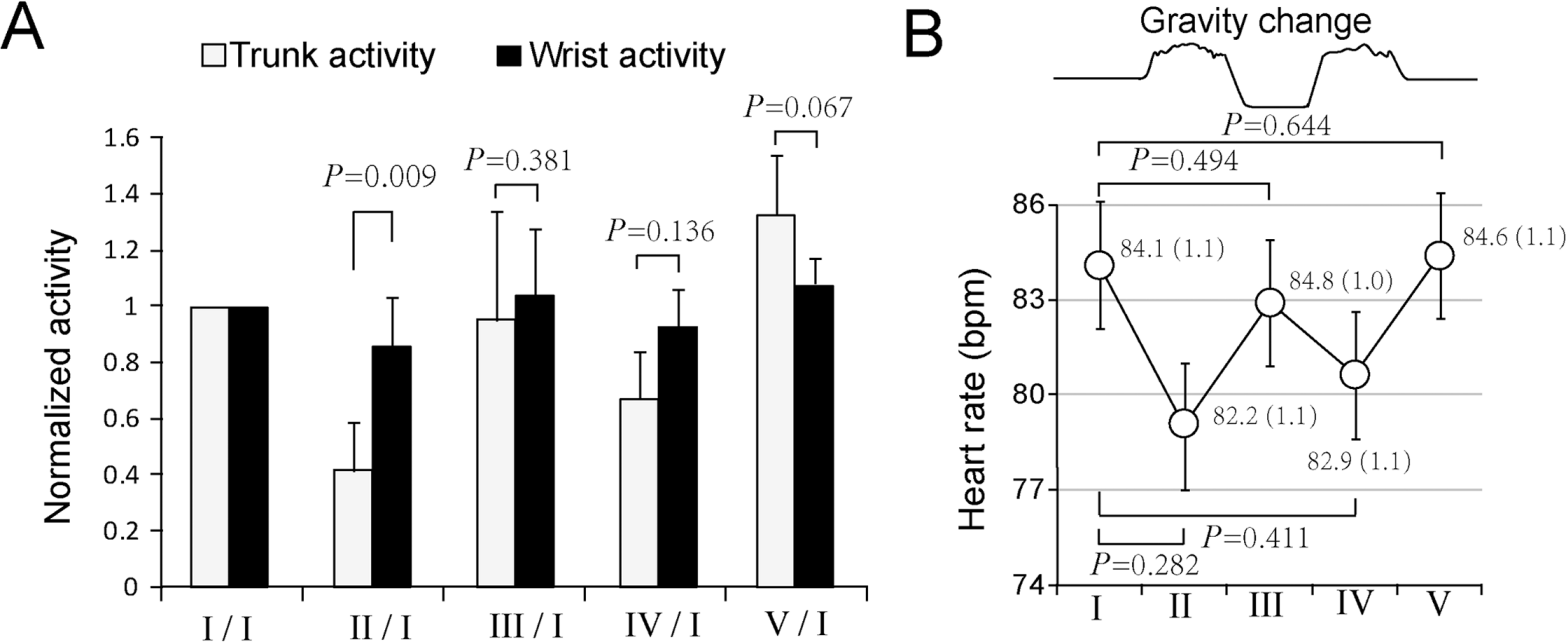
Influences of parabolic flight on activity and heart rate. (A) Changes in trunk and wrist activities during the five time blocks. The data obtained from seven subjects are presented as the average values of seven subjects, mean ± SE. The values in block I were normalized to 1.0. Statistical significance was determined by Student-*t* test. Data are mean ± SE, n = 7. (B) The heart rates during the five time blocks. The data obtained from seven subjects are averages of all tests completed by eight subjects. Statistical significance was determined with one-way repeated-measures ANOVA with Fisher’s least significance difference (LSD) post hoc test. Data represent mean ± SE, n = 195.The data are means ± SEs. The *P* values are shown to indicate significant differences.

To further compare the influence of parabolic flights on wrist and trunk activities, the average values of the activity data from phase II-V for the seven subjects were normalized to the activities during phase I. The data from one subject were precluded since the averaged values at phase I was 0 (Table 1). The results revealed that the trunk activity significantly decreased (0.42 ± 0.17) in phase II only (Fig 2A). The wrist activities showed no significant change across all the phases. Together, these data suggest that hypergravity conditions might lead to reductions in trunk activity.

### Effects of parabolic flight on heart rate

Altered gravity causes physiological changes in the cardiovascular system that include heart rate and blood pressure [11, 21, 22]. The Actihearts were used to record both the trunk activity and heart rate data. The heart rate results of the 195 person-time parabolas demonstrated that, compared to phase I, the heart rates were decreased at phases II and IV and increased at phases III and V (Fig 2B, Table 2), and these changes displays a W- shaped pattern. However, none of these changes reached statistical significance.

**Table 2:** Changes in heart rate during the parabolic flights.

Subjects	Heart rate				
	Parabolic phases				
	I	II	III	IV	V
**1**	78.9 (1.6)	73.4 (1.1)	86.8 (1.6)	80.9 (1.5)	82.5 (2.0)
**2**	81.8 (3.0)	77.6 (1.6)	74.7 (1.2)	79.7 (2.6)	82.0 (2.9)
**3**	88.9 (2.0)	83.7 (2.5)	79.2 (1.9)	83.0 (2.3)	88.2 (2.6)
**4**	84.0 (2.1)	76.4 (1.8)	85.7 (1.8)	74.7 (1.5)	79.9 (2.2)
**5**	72.5 (1.8)	81.9 (4.9)	84.4 (3.6)	81.8 (3.0)	78.5 (2.3)
**6**	108.4 (2.1)	105.6 (3.6)	104.7 (3.1)	105.5 (3.5)	109.7 (2.4)
**7**	80.6 (2.1)	87.4 (2.8)	87.3 (2.6)	84.0 (2.5)	78.9 (2.5)
**8**	58.0 (1.3)	57.3 (0.9)	60.8 (3.0)	57.3 (2.0)	62.0 (1.8)

The data are represented as the average of heart rate during all the parabolas of each subject. Some data containing unreasonable values of heart rate (e.g., 0) due to the loose belt of Actiheart, were considered invalid and precluded for analysis. Data are means (SEs).

### Influence of water immersion on wrist and trunk motions

**Fig 3:**
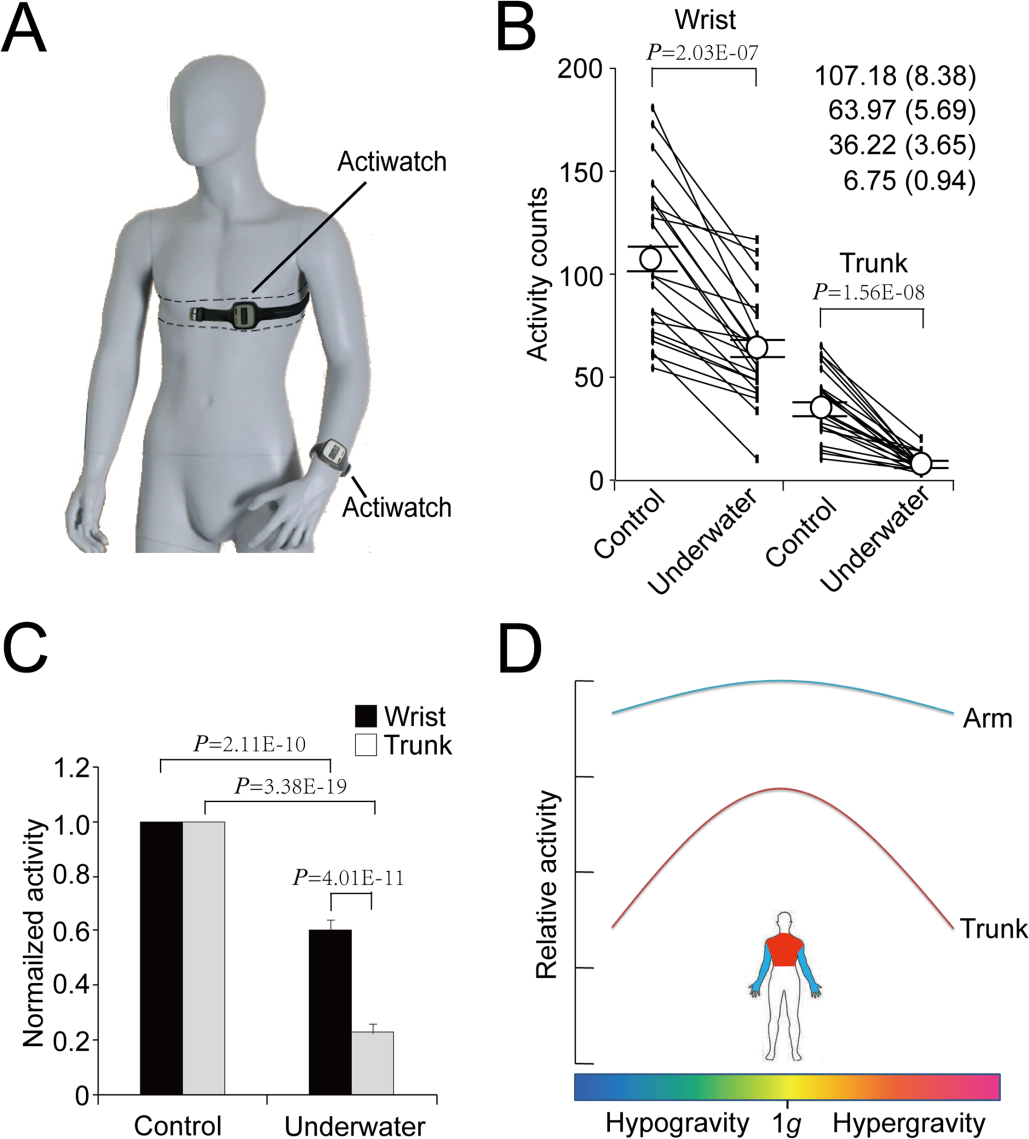
Influence of water immersion on wrist and trunk activities. (A) The placement of the Actiwatchs on a human subject. The Actiwatch on the chest was sealed in double-layered plastic bag and wrapped with bandage around chest along the dashed lines. (B) Wrist and trunk activities recorded by the Actiwatchs. The data represent means ± SEs, n = 22. (C) Changes in the trunk and wrist activities. The data are means ± SEs, n = 22. The values in block I were normalized to 1.0. Statistical significance was determined with Student-*t* test. (D) Schematic presentation of the changes in motion in the different gravity conditions.

The subjects wore Actiwatchs on their wrists and chests simultaneously to record the wrist activity and trunk data. The data from 22 person-time experiments completed by 11 subjects revealed that in comparison to the control data collected on land, both the wrist and trunk activities significantly decreased (Fig 3B, Table 3). After normalization, the relative activity of the wrist dropped to be 0.61 ± 0.04, and that of the trunk decreased to 0.23 ± 0.03. The latter decrease was significantly greater than the former (Fig 3C), suggesting that the gravity reduction simulated by water immersion differentially affected the motions of the trunk and wrist.

**Table 3:** Changes in wrist and trunk motions on the ground and underwater.

Experiments.	Control		Underwater	
	Wrist motion	Trunk motion	Wrist motion	Trunk motion
1	41.35 (2.10)	94.80 (4.63)	6.29 (0.43)	43.72 (4.29)
2	180.87(10.07)	43.77 (4.18)	70.56 (7.17)	4.38 (0.77)
3	58.62 (2.52)	123.85 (1.98)	4.25 (2.33)	55.71 (1.95)
4	81.71 (6.27)	30.55 (3.47)	33.81 (3.71)	3.832 (0.78)
5	134.04 (9.95)	25.35 (2.53)	64.58 (4.46)	6.08 (1.69)
6	81.84 (6.67)	32.02 (4.04)	60.27 (3.86)	6.59 (0.71)
7	124.25 (10.29)	60.98 (8.40)	51.56 (5.07)	6.75 (0.91)
8	69.93 (7.29)	23.65 (3.20)	48.54 (4.85)	13.16 (2.48)
9	172.91 (14.76)	60.78 (6.32)	102.22 (4.71)	18.97(1.52)
10	33.26 (13.61)	127.39 (4.40)	6.22 (9.45)	116.91 (0.93)
11	63.55 (6.45)	13.72 (2.52)	10.30 (2.57)	1.97 (0.67)
12	99.24 (7.50)	12.25 (1.99)	83.83 (5.17)	4.35 (0.74)
13	60.25 (5.74)	9.43 (1.69)	42.48 (3.77)	3.95 (0.69)
14	54.53 (6.75)	25.49 (4.03)	39.73 (4.99)	2.02 (0.44)
15	76.87 (6.78)	27.15 (3.05)	68.23 (4.948)	13.64 (1.322)
16	72.11 (7.65)	15.72 (2.54)	52.40 (4.21)	6.35 (0.83)
17	161.74 (10.07)	65.41 (4.50)	93.37 (6.59)	5.28 (0.69)
18	136.21 (10.51)	44.48 (4.20)	63.10 (4.96)	12.68 (3.05)
19	144.05 (8.88)	54.13 (3.95)	80.38 (5.93)	5.26 (0.71)
20	132.74 (14.50)	32.84 (3.65)	110.73 (7.31)	7.73 (1.05)
21	99.17 (7.67)	43.03 (4.96)	64.35 (6.05)	6.19 (1.46)
22	67.55 (6.32)	42.67 (4.12)	48.46 (3.66)	2.03 (0.36)
Mean (SE)	107.18 (8.38)	36.22 (3.65)	63.97 (5.69)	6.75 (0.94)

The data represent the average of wrist and trunk activity counts of each subject. Data are means (SEs).

## Discussion

Gravitational alterations exert prominent impacts on the cardiovascular system due to the redistribution of body fluid, and changes in gravity elicit profound cardiovascular effects in animals and humans [11, 22–26]. Beckers et al. reported that during parabolic flights, subjects’ heart rate display significant variability while the subjects are in the standing position [23]. In contrast, heart rate values recorded in the supine position exhibit no significant differences between the different phases [23]. These results suggest that the position affects the influence of gravity on heart rate because the supine position can attenuate the redistribution of body fluid caused by gravity change [2]. Previous studies have used electrocardiogram (ECG) monitors to record heart rate [4, 23]. Beckers et al. also reported that in the standing position, the heart rate RR interval (inverse of heart rate) is decreased in hypogravity and increased in microgravity. In contrast, the supine position, the cardiovascular effects of hypergravity are compromised and exhibit no significant influence on heart rate variability [23]. In this work, we analyzed the changes that occurring during parabolic flights and in agreement, our study revealed that squatting or supine position minimized the effects of gravity alterations on heart rate. Interestingly, mental arithmetic also seems to be effective in altering cardiovascular activity during parabolic flight [27–29].

In a previous study, we reported a dramatic decrease in the trunk activities in two orbital astronauts [17]. To validate these findings, in the present work, we analyzed the wrist and trunk activity data collected from parabolic flights and water immersion experiments. The data from parabolic flights indicated that the trunk motion counts dropped by approximately half in the hypergravity condition (phase II) (Fig 2A). Unlike the microgravity condition of the space mission [16], the trunk activities exhibit no significant changes in the microgravity during parabolic flights (phase III) (Fig 1C and Fig 2A). This inconsistency might be attributed to combined influences of the different phases and the short durations during each phase. The water immersion results demonstrated that in the underwater condition, the trunk motion dropped to a level that was significantly lower than that of the wrist (Fig 3A). Together, these data suggest that gravitational changes lead to decreases in trunk motion (Fig 3C). De Witt et al. investigated the changes in motion patterns in actual and simulated weightless conditions. Although there was no direct comparison of the locomotion patterns, including trunk motion, between the conditions of altered gravity and 1 *g* gravity, the differences in motion patterns during low and high degrees of actual and simulated weightlessness were observed [29]. For instance, in a single walking stride, the ankle angle was increased while decreased in the angles of hip, knee and ankle in running and ankle angle in the simulated low gravity condition. The hip angle was increased in actual low weightlessness during both walking and running conditions [29]. The changes in hip kinematics may reflect decreases in trunk motion in simulated weightlessness [29].As such, despite the inconsistencies between simulated and actual weightless, these data support our findings of the present work.

It is necessary to mention that the differences between the activities performed in 1.8*g* condition and other conditions during the parabolic flights, and between the underwater and on-land conditions might not be equivalent, which is an additional possibility that could in part explain the changes in trunk motion. However, the consistency of the data derived from the orbital study [17], and the parabolic flights and water immersion experiments described here, suggest that the gravitational changes are the predominant explanation. Hypogravity and water immersion can result in reduction in weight loads, which might account for decreases in trunk movement because in such conditions, many activities require less strength [17]. In contrast, it is likely that in the hypergravity condition, trunk motion is reduced to withstand the increased gravity. Therefore, hypogravity and hypergravity might lead to decreases in trunk motion for different reasons (Fig 3C). Wearing the Actiheart causes discomfort for females because the Actiheart has to be worn tightly below the breasts, we did not include female subjects in this work.

Muscle cell atrophy is one of the primary problems associated with weightlessness. An analysis of the changes in the locomotor systems of the astronauts aboard Skylab and Mir showed that the mass loss in leg extensors and that these reductions occurred more rapidly than those in the flexors during a short-termed space mission, but both groups of muscles displayed similar declines in isokinetic strength of approximately 30%when the flight duration was sufficiently long (>200 days) [30].An analysis o data collected during an 8-day spaceflight showed that greater mass losses occurred in the intrinsic back muscles than in the soleus-gastrocnemius, anterior calf, hamstrings and quadriceps [15].The prominent mass loss of the back muscles is consistent with the significant decrease in trunk motion, although whether it accounts for more rapid trunk muscle loss remains to be determined. It will be necessary to employ multiple approaches to extensively compare the physiological changes that occurring in different body parts in altered gravity conditions.

In space, astronauts suffer from back pain that might hinder their abilities to perform challenging tasks. Indeed, in the space station, a substantial portion of space medicine is dedicated to the treatment of back pain and motion disorders [31]. Expansion of the lumbar intervertebral discs due to decreased gravitational compression might account for this back pain [32]. In space, a more than four-fold increase is the occurrence of intervertebral disc herniation [32,33]. Based on the results of bed rest experiment [34], it is likely that movement restrictions also influence the aspects intervertebral discs, including the disc volume, the anterior and posterior disc height and the lordosis angle. The influence of bed rest on intervertebral discs may be attributable to the loss of gravitational load on the trunk and movement confinement. Therefore, the decreases in the activities of the trunk muscles that occur during weightlessness might aggravate the expansion of the lumbar intervertebral discs.

Moreover, the postures and orientation abilities of humans and a variety of animals are altered in migrogravity, and weight loads can reverse the postural reactions to microgravity [12,35,36]. The reduction in trunk motion might also be associated with postural and orientational changes. Thus, certain countermeasures, including the application of compensatory load forces to the trunk and exercise of the trunk muscles, should be considered to minimize the detrimental effects.
